# Paediatric palliative care by video consultation at home: a cost minimisation analysis

**DOI:** 10.1186/1472-6963-14-328

**Published:** 2014-07-28

**Authors:** Natalie K Bradford, Nigel R Armfield, Jeanine Young, Anthony C Smith

**Affiliations:** 1 Centre for Online Health, University of Queensland, Level 3 Foundation Building Royal Children’s Hospital, Herston Rd, Herston, QLD 4029, Australia; 2 Queensland Children’s Medical Research Institute, University of Queensland, Royal Children’s Hospital, Herston Rd, Herston, QLD 4029, Australia; 3 School of Nursing and Midwifery, University of the Sunshine Coast, Sippy Downs, QLD 4556, Australia

**Keywords:** Economic evaluation, Palliative care, Home telehealth, Health services research

## Abstract

**Background:**

In the vast state of Queensland, Australia, access to specialist paediatric services are only available in the capital city of Brisbane, and are limited in regional and remote locations. During home-based palliative care, it is not always desirable or practical to move a patient to attend appointments, and so access to care may be even further limited. To address these problems, at the Royal Children’s Hospital (RCH) in Brisbane, a Home Telehealth Program (HTP) has been successfully established to provide palliative care consultations to families throughout Queensland.

**Methods:**

A cost minimisation analysis was undertaken to compare the actual costs of the HTP consultations, with the estimated potential costs associated with face-to face-consultations occurring by either i) hospital based consultations in the outpatients department at the RCH, or ii) home visits from the Paediatric Palliative Care Service. The analysis was undertaken from the perspective of the Children’s Health Service. The analysis was based on data from 95 home video consultations which occurred over a two year period, and included costs associated with projected: clinician time and travel; costs reimbursed to families for travel through the Patients Travel Subsidy (PTS) scheme; hospital outpatient clinic costs, project co-ordination and equipment and infrastructure costs. The mean costs per consultation were calculated for each approach.

**Results:**

Air travel (n = 24) significantly affected the results. The mean cost of the HTP intervention was $294 and required no travel. The estimated mean cost per consultation in the hospital outpatient department was $748. The mean cost of home visits per consultation was $1214. Video consultation in the home is the most economical method of providing a consultation. The largest costs avoided to the health service are those associated with clinician time required for travel and the PTS scheme.

**Conclusion:**

While face-to-face consultations are the gold standard of care, for families located at a distance from the hospital, video consultation in the home presents an effective and cost efficient method to deliver a consultation. Additionally video consultation in the home ensures equity of access to services and minimum disruption to hospital based palliative care teams.

## Background

The need for specialist paediatric palliative care is rare; in developed nations approximately only 15 in 10’000 children aged 0–19 will require such services [1]. Children who require palliative care may be dispersed throughout urban, regional and rural areas. Families caring for a child at the end of life, require timely support and competent care. However, many families choose to care for their child in their home location [2], and may not live in an area where specialist Paediatric Palliative Care Services (PPCS) are available [3].

To address this, in 2009 a PPCS was established in Queensland, Australia, which routinely uses telehealth to communicate with families and primary care clinicians who are dispersed across the state. As well as using standard hospital-based videoconferencing systems to connect with regional facilities, a Home Telehealth Program (HTP) was established using Internet video-links to facilitate a specialist PPCS consultation directly into families’ homes. While the accepted gold-standard of care is a face-to face consultation, where it is impractical for this to occur, the HTP is able to facilitate a consultation, ensuring a family is not disadvantaged by location. The effectiveness of the HTP in increasing equity of access to care has been previously described elsewhere [4].

Understanding the economic effects of the HTP is also important. Other economic evaluations for telehealth services have not always identified savings to the health service [5]. Health services are required to make choices regarding the best way to use limited resources to provide services; therefore economic evaluations are becoming increasingly important in providing evidence to inform these decisions.

The aim of this cost minimisation analysis was to compare the costs of paediatric palliative care medical consultations conducted via the HTP with in-person consultations. This evaluation is provided from the perspective of the savings to the Children’s Health Service, a state-wide service and assumes comparable outcomes irrespective of how services were delivered.

## Methods

### Analytical approach

Permission was obtained from Queensland Health PPCS director, and approval granted from the Royal Children’s Hospital Human Research Ethics Committee (reference number HREC/03/QRCH/16 AM02) to access the PPCS patient database. Records were retrieved from the database to identify the number of HTP consultations that had occurred over a 24-month period (July 2009 - June 2011). Data collected included the location of the family, participants and duration of the consultation. The actual costs of providing the HTP were compared to potential costs if in-person consultations had occurred. Outcomes were assumed to have been equivalent whether the consultation had occurred in person or via the HTP. The methods used in this analysis have been reported in other telehealth studies and are described in full for the benefit of other researchers [6-9]. Usual care consultations for outpatients occur in-person either at the hospital outpatients department (OPD) or when the PPCS visit the patient’s home. A cost minimization analysis was undertaken to compare these three possible approaches:

A. HTP consultation (intervention)

B. Home visit consultation (usual care)

C. OPD consultations (usual care)

The fixed and variable costs for all three approaches were determined. Fixed costs were those independent of the number of consultations that occurred. Variable costs were those associated with the activity of the program and therefore vary depending on the number of consultations. Marginal costs were calculated by taking the sum of all variable costs and dividing the total variable costs by the number of consultations [10].

Costs were obtained for the HTP consultations by calculating the fixed and variable costs of providing the HTP over the 24-month study period. Data was obtained for the comparator groups by estimating the costs that would have been incurred by the health service, based on the resource implications of an OPD appointment or home visit consultation.

The model included both fixed and variable costs associated with the HTP (equipment, staff costs) and estimated costs for in-person consultations (travel expenses, clinician’s time, hospital resources). Costs were sourced for resources from departmental records and data available from publically accessible web sites [11-19], and all cost are reported in Australian Dollars.

### Home telehealth program valuation

#### Valuation of fixed and variable equipment and infrastructure costs

The HTP equipment has been previously described [4]. Fixed costs at the hospital end included: telecommunications, a computer and a web camera, which were purchased specifically for the HTP. Conversion to an annual equivalent cost (AEC) was made by annuitizing the cost of equipment over the expected life (3 years) using an annual discount rate of 5%. The AEC was then multiplied by the duration of the study period (24 months) to obtain the attributed cost. All equipment was valued according to its original purchase price to the HTP in 2009. Variable costs included clinician’s time to undertake the consultations and the supply of web cameras to families who required one.

The costs to families for equipment and Internet were not included in the valuation as these items were already available in the household and were not specifically purchased in order to access the HTP.

#### Staff costs

A project officer was employed on a casual basis three days per week (0.6 Full Time Equivalent (FTE). As well as other duties, subject to demand, the project officer set up, organised and facilitated the HTP consultations. These activities involved:

•establishing individual family’s requirements

•providing instructions on downloading, installing and running software

•creation of accounts and passwords

•test video-calls with the family

•co-ordination with clinicians and family regarding date and time for consultations

•documentation of video-consultation summary

HTP consultations occurred approximately once per week and an estimated 10% of the project officer’s time was allocated this. Therefore 6% of FTE was allocated as a fixed cost to establish the HTP (1 FTE = $81,000, 0.06 FTE = $4860 per annum).

To enable a comparison between all three models, it was assumed the same clinicians (PPCS Pediatrican and Nurse Consultant) were present for each HTP consultation, and that each consultation went for 30 minutes duration, the expected duration of an outpatient consultation. Clinician’s time costs were based on Queensland Health hourly rates [12,13] and were calculated to be $123 per 30-minute consultation.

### Valuation of outpatient consultation

#### Cost of appointment

The cost of outpatient appointments at the RCH was based on the Australian National Efficient Price (NEP) as a proxy for costs. The NEP is determined by the Independent Hospital Pricing Authority through analysis of data on activity and costs in Australian public hospitals, and provides a set amount for each activity or service. For a paediatric medical outpatient consultation (code 20.11) this equates to $351 per consultation [11]. This includes the direct costs of clinician’s time, and also the indirect costs associated with hospital based consultations such as administration, laundry and cleaning etc. As the NEP price is assumed to include clinician’s time, the calculation for both the PPCS Paediatrician and Nurse Consultant is not included in the valuation of the outpatient appointment.

#### Patient and caregiver travel

For travel costs associated with attending a hospital outpatient consultation, it was assumed that travel would have occurred by car for locations less than 300 km from the RCH. For locations greater than 300 km it was assumed that the child and caregiver would travel by air to attend the RCH and require accommodation for one night. These travel costs are subsidised by the health system through the Patient Travel Scheme (PTS) for patients who reside further than 50 km from the hospital; at the time of the study, families were reimbursed $0.15/km travelled by road, and the full cost of air transport [19]. Estimates of airfares were obtained by Internet search of the cheapest economy airfare fares [17]. Accommodation was subsided at $30 per person per night and was included in the analysis in cases where travel by air is assumed to have occurred. Locations of all families who had HTP consultations during the study period were obtained and the costs to attend the RCH OPD were calculated for each location.

#### Additional costs

Other cost minimisation studies have reported additional costs to families such as time off work, child care arrangements and meals [7]. As all primary caregivers in this study had given up full time work to care for their child, these expenses were not included.

### Valuation of home-visit consultation

The duration of a consultation was assumed be identical between HTP and in-person consultations (30 minutes). The extra time required for travel for a home visit was calculated. To calculate the assumed costs for home visits, the same locations of families used in the calculations for OPD consultations. Calculations were based on the same clinicians attending a home visit as in the HTP consultations, i.e. the PPCS paediatrician and the PPCS nurse consultant. Clinician time costs were based on Queensland Health hourly rates [12,13]. It was assumed that for distances less than 300 km the clinicians would travel by road to the patient’s home, and for distances greater than 300 km travel by air. Travel by air included estimated costs for return clinician travel time and transport (airfare, taxi fare) as well as accommodation for one night (valued at $150 per person) for two visiting clinicians. Airfares were calculated the same way as patient and caregiver travel costs and taxi fares were calculated using an online taxi fare calculator [18].

### Economic analysis

Understanding the effect each variable has on the overall costs and the best efficient price is important for decision makers and health services. The threshold point was calculated to determine the point at which the cost of the HTP was equal to the cost of either alternative service model.

The total costs of each approach were calculated by summing all fixed and variable costs. As it is acknowledged that it is not practical or feasible for air travel to occur for only a single consultation, this analysis was also completed for the costs associated with travel by road only. The costs of both options are presented separately. A sensitivity analysis was used to examine the decision analytical models and determine the effects of varying a particular cost. The sensitivity analysis therefore is able to provide an assessment that takes into account the costs that may differ from the assumed costs in the broader analysis. The sensitivity analysis was undertaken using the total of all consultations, i.e. both road and air travel.

The total cost of the HTP was:

$$\mathrm{HTP}=F_{\mathrm{HTP}}+xV_{\mathrm{HTP}}$$

Where:

F_*HTP*_ is the fixed cost of the HTP

*x* is the number of consultations

V_*HTP*_ is the marginal cost per HTP consultation

The threshold (*x*) for OPD consultations occurred when:

$$\begin{array}{l} \mathrm{Total}\;\mathrm{cost}\;\mathrm{of}\;\mathrm{HTP}=\mathrm{Assumed}\;\mathrm{total}\;\mathrm{cost}\;\mathrm{of}\;\mathrm{OPD}\;\\ \;\;\mathrm{consultation}\\ \;F_{\mathrm{HTP}}+xV_{\mathrm{HTP}}=F_{\mathrm{OPD}}+xV_{\mathrm{OPD}} \end{array}$$

Where:

F_*OPD*_ is the fixed costs of OPD consultations

V_*OPD*_ is the marginal costs per OPD consultation

The threshold (*x*) for home visit consultations occurred when:

$$\begin{array}{l} \mathrm{Total}\;\mathrm{cost}\;\mathrm{of}\;\mathrm{HTP}=\mathrm{Assumed}\;\mathrm{total}\;\mathrm{cost}\;\mathrm{of}\;\mathrm{Home}\\ \;\;\mathrm{visit}\\ \;F_{\mathrm{HTP}}+xV_{\mathrm{HTP}}=F_{\mathrm{HV}}+xV_{\mathrm{HV}} \end{array}$$

Where:

F_*HV*_ is the fixed costs of home visits by the PPCS

V_*HV*_ is the marginal costs per home visit

The following formula was used for the calculation of the threshold point (*x*) for comparison of HTP and OPD appointments:

$$\begin{array}{l} F_{\mathrm{HTP}}–F_{\mathrm{OPD}}=xV_{\mathrm{OPD}}–xV_{\mathrm{HTP}}\\ F_{\mathrm{HTP}}–F_{\mathrm{OPD}}=x\left(V_{\mathrm{OPD}}–V_{\mathrm{HTP}}\right)\\ \;x=\left(F_{\mathrm{HTP}}–F_{\mathrm{OPD}}\right)/\left(V_{\mathrm{OPD}}–V_{\mathrm{HTP}}\right) \end{array}$$

The same formula was used to calculate the threshold point for comparison of HTP and home visit consultations.

$$\begin{array}{l} F_{\mathrm{HTP}}–F_{\mathrm{HV}}=xV_{\mathrm{HV}}–xV_{\mathrm{HTP}}\\ F_{\mathrm{HTP}}–F_{\mathrm{HV}}=x\left(V_{\mathrm{HV}}–V_{\mathrm{HTP}}\right)\\ \;x=\left(F_{\mathrm{HTP}}–F_{\mathrm{HV}}\right)/\left(V_{\mathrm{HV}}–V_{\mathrm{HTP}}\right) \end{array}$$

## Results

### Actual cost of the home telehealth program

Analysis of the PPCS database yielded a total of 95 clinical consultations during the study period, 24 were at distances greater than 300 km from the RCH and 71 within 300 km of the RCH. The locations of patients, distance from the RCH and number of consultations undertaken in each location are presented in Table 1.

**Table 1:** Location of patients, distance from RCH and number of consultations undertaken

**District**	**Distance from RCH (km)**	**Number of consultations (n)**
Brisbane	10	24
Ipswich	43	5
Redcliffe	28	11
Moggill	20	3
Sunshine Coast	88	11
Gold Coast	96	12
Lismore	187	3
Kingaroy	189	2
Bundaberg	357	2
Emerald	854	1
Cairns	1694	8
Mt Isa	1826	13
***Total number of consultations***		***95***

### Actual costs for the HTP

The total cost for the HTP consultations over the 24-month period are presented in Table 2. While many families had their own equipment including web cameras, during the study period 10 families were provided with a web camera valued at $30 each. For the 95 consultations over 24 months, the mean cost per consultation was calculated to be $251, with a marginal cost of $126.

**Table 2:** Total costs of the HTP over 24 months for 95 consultations

**Resources**	**Actual cost $**
**Fixed set up costs**	
Videoconferencing equipment*	$1144
ADSL line rental	$990
Co-ordinator	$9720
*Sub Total*	*$11,854*
**Variable ongoing costs**	
Clinician consultation time^#^	$11,685
Web cameras for families	$300
*Subtotal*	*$11,985*
***Total***	***$23,839***
*Mean cost per consultation*	*$251*
*Marginal cost per consultations*	*$126*

*proportion of costs over 24 months annuitized over three years.

^#^clinician consultation time includes PPCS paediatrician and nurse consultant for 30 minutes.

### Assumed costs of OPD consultations

Costs associated with patient and caregiver attendance at the RCH OPD are presented in Table 3. The mean cost of an OPD consult was $748. This result was significantly affected by consultations assumed to have required air travel. Where air travel was assumed to have occurred (n = 24), the cost of attending an OPD appointment was calculated at a mean cost of $1903. Where travel by road only was assumed to have occurred (n = 71) the mean cost was reduced to $357.

**Table 3:** Assumed costs of OPD consultations (n = 95)

**District**	**A**	**B**	**C**^**#**^	**D**	**E**
	**(n)**	**($)**	**($)**	**($)**	**($)**
**Travel by Road**					
Brisbane	24	$8424	N/A	-	$8424
Ipswich	5	$1755	N/A	-	$1755
Redcliffe	11	$3861	N/A	-	$3861
Moggill	3	$1053	N/A	-	$1053
Sunshine Coast	11	$3861	$13	$143	$4004
Gold Coast	12	$4212	$14	$168	$4380
Lismore	3	$1053	$22	$66	$1119
Kingaroy	2	$702	$31	$63	$765
*Subtotal road travel*	*71*	*$24,921*		*$440*	$*25,361*
**Travel by air**					
Bundaberg	2	$702	$1158	$2316	$3018
Emerald	1	$351	$1542	$1542	$1893
Cairns	8	$2808	$1602	$12,816	$15,624
Mt Isa	13	$4563	$1582	$20,566	$25,129
*Subtotal air travel*	*24*	$*8424*		*$37,240*	$*45,664*
***Total***	***95***	***$33345***		***$37,680***	***$71,025***
*Mean $ road travel*					*$357*
*Mean $ air travel*					*$1903*
*Mean $ all travel*					*$748*

#PTS subsidy 15c per km, all airfares and accommodation subsidy $30 per person when more than 50 km from RCH [19].

A. Number of consultations.

B. Cost of consultations (A × $351).

C. Cost per return trip for child and parent #.

D. Travel cost (A × C).

E. Total cost to health service (B + D).

### Assumed costs of home visit consultations

Costs associated with PPCS home visits are summarised in Table 4. The mean cost estimated for a home consultation by the visiting PPCS was $1214 (n = 95). This cost was also significantly affected by the cases that were assumed to have required air travel. Where travel by air is assumed to have occurred (n = 24) the mean costs was $3303. Where travel by road only (n = 71) is assumed to have occurred, the mean cost was estimated at $507.

**Table 4:** Assumed costs of home visits (n = 95)

**Total Costs**	**A**	**B**	**C**	**D**
	**Clinician consultation time**	**Clinician travel time**	**Clinician Travel costs**	**Total cost (A + B + C)**
	**$**	**$**	**$**	**$**
Brisbane	$2952	$3648	$72	$6672
Ipswich	$615	$1645	$120	$2380
Redcliffe	$1353	$2783	$110	$4246
Moggill	$369	$759	$21	$1149
Sunshine Coast	$1353	$6123	$319	$7795
Gold Coast	$1476	$7284	$408	$9166
Lismore	$369	$2429	$141	$2939
Kingaroy	$246	$1265	$140	$1651
*Subtotal road travel*	$8733	$25,936	$1403	$35,998
**Travel by air n = 24**				
Bundaberg	$246	$2530	$4182	$6958
Emerald	$123	$1695	$1808	$3626
Cairns	$984	$11,132	$14,736	$26,852
Mt Isa	$1599	$15,787	$24,466	$41,852
*Subtotal air*	$2952	$31,144	$45,192	$79,288
***Total***	**$11,685**	**$** ***57,080***	**$** ***46,595***	**$** ***115,286***
*Mean $ road travel*	*$123*	*$365*	*$20*	*$507*
*Mean $ air travel*	*$123*	*$1298*	*$1883*	*$3,303*
*Mean $ all travel*	*$123*	*$601*	*$490*	*$1214*

### Summary of actual and estimated costs

The fixed and variable costs of providing the HTP over a 24 month period and the estimated costs of providing the same consultations via OPD visits or home visits are presented in Table 5. In Table 6, the data is presented for those consultations which are assumed to have required road only travel.

**Table 5:** Actual costs of HTP program and assumed costs for OPD or home visits for 95 consultations (all modes of travel)

**Expenditure**	**A**	**B**	**C**
	**HTP consultation**	**OPD consultation**	**Home visit consultation**
	**$**	**$**	**$**
**Fixed costs**			
Equipment (3 year total annuitized cost)	$1144		
ADSL line rental	$960		
Co-ordinator	$9720		
*Sub total*	$*11,824*		
**Variable costs**			
Web cameras	$300		
PTSS costs		$37,680	
Cost of ODP consultation		$33,345	
Clinician consultation time	$11,685	*	$11,685
Clinician travel time	-	-	$57,080
Clinician travel costs			$46,595
*Sub total*	$11,985	$*71,025*	$*115,360*
***Total cost***	**$23,509**	**$71,025**	**$115,360**
*Mean cost*	*$247*	$*748*	$*1214*
*Marginal cost*	*$123*	*$748*	*$1214*

*included in cost of OPD consultation.

**Table 6:** Actual costs of HTP program and assumed costs for OPD or home visits for 71 consultation (road only travel assumed)

**Expenditure**	**HTP consultation**	**OPD consultation**	**Home Visit Consultation**
	**$**	**$**	**$**
**Fixed costs**			
Equipment (3 year total annuitized cost)	$1144		
ADSL line rental	$960		
Co-ordinator	$9720		
*Sub total*	$*11,824*		
**Variable costs**			
Web cameras	$300		
PTSS cost		$440	
Cost of ODP consultation		$24,921	
Clinician consultation time	$8733	*	$8733
Clinician travel time			$25,936
Clinician travel costs			$1403
*Sub total*	$9033	*$25,364*	*$36072*
***Total costs***	***$20,857***	***$25,364***	***$36072***
*Mean cost*	*$294*	*$357*	*$508*
*Marginal cost*	*$123*	*$357*	*$508*

*included in cost of OPD consultation.

For a one-year period this equates to:

A. HTP consultations = $11,755

B. OPD consultations = $35,513

C. Home visit consultations = $57,680

For a one year period, for consultations assumed to require road only transport (i.e. those consultations less that 300 km distance from the RCH), this equates to:

A. HTP consultation = $10,438

B. OPD consultation = $12,682

C. Home visit consultation = $18,036

### Statistical analysis

#### Threshold analysis

Using the calculations from Table 5, the threshold point was calculated for all consultations.

For this evaluation, the threshold point for OPD consultations was:

$$\begin{array}{l} x=\left(F_{\mathrm{HTP}}–F_{\mathrm{OPD}}\right)/\left(V_{\mathrm{OPD}}–V_{\mathrm{HTP}}\right)\\ \;=\left(11824-0\right)/\left(748–123\right)\\ \;=11824/625=19 \end{array}$$

For this evaluation, the threshold point for home visit consultations was:

$$\begin{array}{l} x=\left(F_{\mathrm{HTP}}–F_{\mathrm{HV}}\right)/\left(V_{\mathrm{HV}}–V_{\mathrm{HTP}}\right)\\ \;=\left(11824-0\right)/\left(1214–123\right)\\ \;=11824/1091=11 \end{array}$$

**Figure 1:**
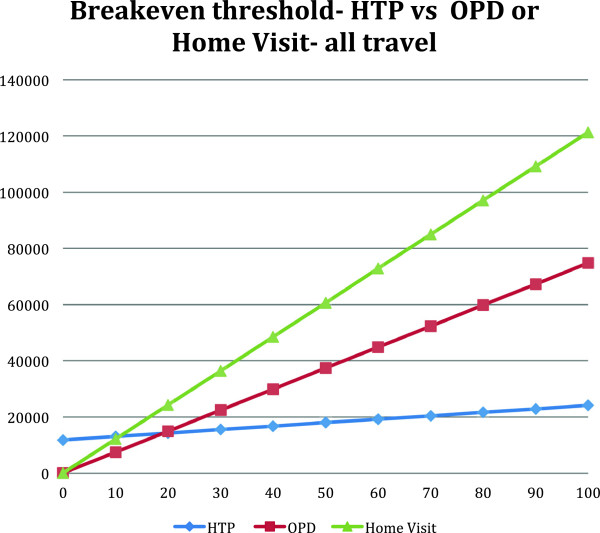
Threshold calculation comparing HTP with OPD or home visit consultations.

The threshold point was reached with 19 patients for HTP consultations versus OPD consultations and at 11 patients for HTP versus home visit consultations (Figure 1).

It was evident that the cost associated with in-person consultations was significantly affected by the need to travel by air. For this reason the threshold calculation was also completed for the costs associated with travel by road only. The data from Table 6 was used to calculate this threshold point.

For this evaluation, the threshold point for road only travel for OPD consultation was:

$$\begin{array}{l} x=\left(F_{\mathrm{HTP}}–\;F_{\mathrm{OPD}-\mathrm{road}\;\mathrm{only}}\right)/\left(V_{\mathrm{OPD}-\;\mathrm{road}\;\mathrm{only}}–V_{\mathrm{HTP}}\right)\\ \;=\left(11824-0\right)/\left(357–123\right)\\ \;=11824/234\\ \;=51 \end{array}$$

For this evaluation, the threshold point for road only travel for home visit consultations was:

$$\begin{array}{l} x=\left(F_{\mathrm{HTP}}–F_{\mathrm{HV}\;\mathrm{road}\;\mathrm{only}}\right)/\left(V_{\mathrm{HV}-\mathrm{road}\;\mathrm{only}}–V_{\mathrm{HTP}}\right)\\ \;=\left(11824-0\right)/\left(508–123\right)\\ \;=11824/385\\ \;=31 \end{array}$$

**Figure 2:**
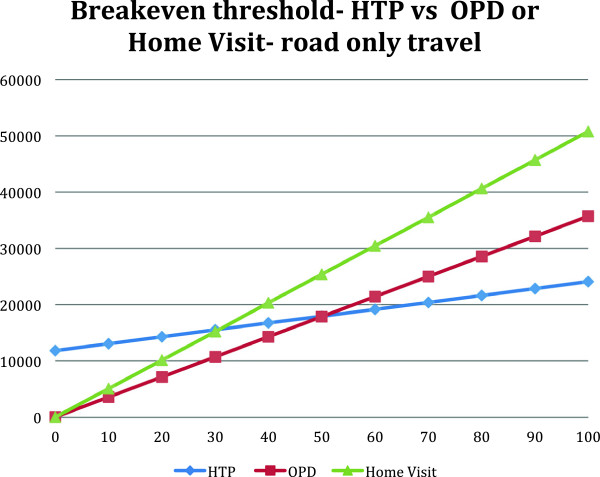
Threshold calculation comparing HTP with OPD or home visit consultationswhere road only travel is assumed.

The threshold point was reached with 51 patients for HTP consultation versus OPD consultations, and by 31 patients for home visit consultations where road travel is assumed to have occurred (Figure 2).

#### Sensitivity analysis

The sensitivity analysis identified the effect of reducing the variable costs associated with OPD and home visit consultations. As it was evident that air travel significantly affected the threshold, undertaking a sensitivity analysis was able to quantify these effects further. Additionally this analysis was able to identify the costs that had the greatest and least effects on the threshold points. As costs of variables are likely to change over time, for example, equipment costs are likely to become less expensive, but costs associated with wages and travel may become more expensive, the analysis was based on all estimated costs. Each unit cost was adjusted by increasing or decreasing its value by 25 and 50%, while holding all other variables constant to assess the effect on threshold. In this analysis the unit costs which had the greatest effect on threshold were the costs associated with wages for co-ordination of the HTP, travel and the cost of OPD consultations. Equipment costs had the least effect on the threshold point. To identify the variable most sensitive to change, the total changes seen in the threshold calculation for each variable were divided by the original baseline threshold calculation. These ratios are expressed as percentages and are listed in order of effect on the threshold point for all three approaches in Table 7.

**Table 7:** All costs listed in order of effect on threshold

**Sensitivity**	**Modality**	**Sensitivity to change ratio (%)**
Co-ordinator salary	HTP consultation	82
Clinician travel time	Home visit	−60
PTSS	OPD consultation	−59
OPD appointment	OPD consultation	−51
Clinician travel costs	Home visit	−47
Clinician consultation costs	HTP consultation	−21
Equipment costs	HTP consultation	10
Clinician consultation costs	Home visit	9
ADSL	HTP consultation	8
Web cameras	HTP consultation	0

## Discussion

The largest costs associated with an in-person PPCS consultation were the cost to the health system of an outpatient consultation or the costs associated with clinician travel. Providing a consultation via the HTP for patients and families less than 300 km from the hospital, where road only travel was assumed, demonstrated a cost saving compared with usual care in both scenarios: compared to delivering consultations via the OPD, the HTP saves the health service $2,244; and compared to delivering the same service via PPCS home visits, the HTP saves the health service $7,598 per annum. When the costs of providing a consultation including those that were assumed to have required air travel are calculated, the cost savings are substantially greater: compared to delivering consultations via the OPD, the HTP saves the health service $23,758 and compared to delivering the same service via home visits, the HTP save the health service $45,925 per annum.

While the assumed costs for families living further than 300 km from the hospital were calculated, in reality it is impractical and highly unlikely that in these cases usual care (i.e. an in-person consultation) would have been possible. The costs associated with air travel make an in-person consultation by usual care expensive; additionally it is not desirable for a child receiving palliative care to travel by air for a simply for one consultation, nor practical for the PPCS to travel by air to visit only one family. The resources required and practicalities of achieving in-person consultation when distance is great, outweighs the benefits.

The HTP is therefore not only an economical method of providing a consultation, but also a practical solution to providing care when a family resides at a distance from the hospital.

The threshold calculations demonstrated that the HTP is cost effective after 19 patients for OPD consultations and only 11 patients for home visits. As 95 patients received a clinical consultation via the HTP during the two year study period, the HTP is an economically feasible and sustainable program, even if fewer patients were seen per annum. This is important to note, as cost effectiveness of telehealth services has been debated [8]. Similar to other studies in Queensland however, this study has identified potential cost savings to the health system related to travel, while providing services closer to patient’s home locations [8,9].

The sensitivity analysis revealed that changes to the costs associated with salaries for the project co-ordinator to co-ordinate the program had more effect on the threshold point than travel or the cost of OPD consultations. Embedding the HTP into the PPCS as a routine part of service delivery could reduce the need for a co-ordinator for the program, further increasing the viability of the HTP as an economical approach to patient care.

This evaluation reflects the minimum saving to the health service that is achievable by using the HTP. The calculation of clinician time for consultations included two clinicians; the PPCS paediatrician and the nurse consultant. The comparator cost for OPD appointments was based on the NEP which accounts for only one clinician’s time, therefore this evaluation is likely to be under estimation of costs avoided. Additionally, since the end of this study period, the Australian government has introduced incentive payments for telehealth consultations as rebates through the Medicare system. These rebates would further support the economic benefits of the HTP for the health system. Other factors not accounted for in these calculations include the presence of other primary health care providers attending the consultation; in 18 (24%) of HTP consultations, multiple health care professionals were in attendance at the family home and participated in the home video-consultation. These additional benefits for undertaking a consultation via telehealth are difficult to value, and have not been included in this analysis, but certainly from a societal perspective the educational opportunity and ability for peer-to-peer support for primary care clinicians are factors that are facilitated by the use of telehealth. While in-person consultations are the deemed gold standard of care, for families located at a distance from the hospital, video-consultation in the home presents an effective and economical method to ensure equity of access to these specialist services.

### Limitations

This study has several limitations. Firstly, all costs for wages for the project co-ordinator and PPCS clinicians have been estimated, as well as costs associated with travel. Additionally the potential additional overhead costs to the RCH for the HTP have not been included. Secondly, the assumption related to travel requirements for patients and clinicians for in person consultations may be atypically of what would actually happen in conventional practice. In some cases, patients in remote locations may have not had access to these services if the telehealth option was not offered. Thirdly, this analysis assumes that equivalent outcomes would have occurred in all three approaches, there may be other benefits and limitations of all three approaches which are not able to be valued.

This analysis however does provide a more accurate reflection of the value of the HTP beyond the costs of the HTP itself. The HTP services a small group of families who require palliative care for their child, and the need for home telehealth services are not likely to increase beyond the activity seen during this study period. While these findings may be generalisable to other services, this study has confirmed that high activity levels are not required for a telehealth service to be economically efficient and sustainable.

## Conclusion

The purpose of this cost minimisation analysis was to compare the actual costs of the HTP with the potential costs of consultations undertaken either by the patient and caregiver attending the RCH for an OPD consultation, or home visits by the PPCS. The analysis demonstrated that based on the activity undertaken over a 24-month period, there were significant costs avoided to the Children’s Health Service. As well as providing an economical approach for delivering a paediatric palliative care consultation, the HTP is able to provide other practical benefits to families and health care clinicians, which are not able to be valued. This study has therefore provided valuable information regarding the effectiveness of the HTP.

## Competing interests

The authors declare that they have no competing interests.

## Authors’ contributions

NB collected the data, undertook the analysis and drafted the manuscript. NR interpreted the data analysis and revised the manuscript. JY participated in the design of the study, its coordination and revised the manuscript. AS conceived of the study, and oversaw the statistical analysis and revised the manuscript. All authors read and approved the final manuscript.

## Pre-publication history

The pre-publication history for this paper can be accessed here:

http://www.biomedcentral.com/1472-6963/14/328/prepub
